# How Long Is Long Enough? Extrapolation of Machine-Learning Interatomic Potentials for Oligomeric and Polymeric Systems

**DOI:** 10.1021/acs.jctc.6c00365

**Published:** 2026-06-19

**Authors:** Natalie E. Hooven, Arthur Y. Lin, Charles H. Carroll, Rose K. Cersonsky

**Affiliations:** † Department of Chemical and Biological Engineering, 5228 University of Wisconsin−Madison, Madison, Wisconsin 53706, United States; ‡ Department of Materials Science and Engineering, 5228 University of Wisconsin−Madison, Madison, Wisconsin 53706, United States; § Data Science Institute, 5228 University of Wisconsin−Madison, Madison, Wisconsin 53706, United States

## Abstract

Machine-learning interatomic potentials (MLIPs) have surged in popularity due to their promise of expanding the spatiotemporal scales possible for simulating molecules with high fidelity. The accuracy of any MLIP is dependent on the data used for its training; thus, for large molecules like polymers and biomolecules, where ab initio training data is prohibitively difficult to obtain, it becomes necessary to use smaller, analogous chemical systems to construct MLIPs. Here, we perform a control study using *n* = 1–8 linear alkanes to determine when MLIPs trained on small molecules can accurately extrapolate to longer chains and more complex architectures. By combining MLIP performance analysis with environment-resolved Smooth Overlap of Atomic Positions descriptors and Principal Covariates Classification, we show that reliable extrapolation emerges when the relevant local chemical environments have converged between the training and target systems. We further demonstrate that careful construction of the neighbor list substantially improves the learnability of intermolecular energetics, the component most critical for polymeric behavior. Together, these results provide a practical, data-driven blueprint for designing transferable MLIPs for macromolecular materials, whether built from bespoke training data or adapted from emerging universal MLIP frameworks.

## Graphical abstract

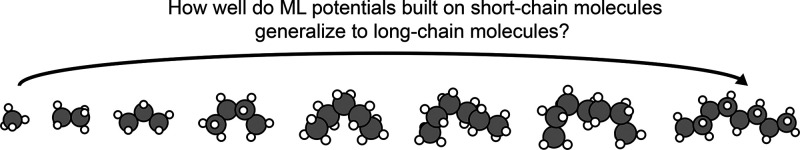

## Introduction

In molecular simulation, the choice of potential energy model determines both the accuracy of predicted behavior and the computational time scales that can be achieved. Classical force fields have played a foundational role in polymer science for decades, enabling the study of thermodynamics, structure, and dynamics across a wide variety of molecular and polymeric systems.^1–3^ Their conceptual clarity, expressing molecular energetics through well-defined bonded and nonbonded terms, combined with their computational efficiency, continues to make them indispensable for simulations that require long time scales, large system sizes, or broad chemical screening.^4,5^

At the same time, advances in electronic-structure theory, machine learning, and high-performance computing have created new opportunities at the interface of accuracy and efficiency. Machine-learned interatomic potentials (MLIPs) offer one such opportunity: by learning from quantum-chemical reference data, they can capture complex many-body interactions without requiring an explicit decomposition into fixed functional forms.^6–13^ MLIPs have recently demonstrated strong performance across problems ranging from ionic transport,^14,15^ condensed-phase phenomena,^16,17^ electronic properties,^18^ and chemical reactivity,^19,20^ motivating interest in when and how these models can complement existing force-field approaches—particularly for systems where nonadditive electronic effects or chemical heterogeneity play an important role.

However, using MLIPs for large molecules such as polymers or biomolecules remains challenging. High-quality quantum chemical data are difficult to obtain for such macromolecules,^21^ and transferability across chain length or architecture is not guaranteed or well-understood. This has led to increasing interest in strategies where smaller, chemically related molecules act as surrogates for constructing potentials that are then applied to larger targets.^22–24^ Although intuitive, the limits of such extrapolation have not been systematically quantified.

Universal or “foundational” machine-learned interatomic potentials (UMLIPs) have recently emerged as a promising direction, offering broad chemical coverage from large, diverse training sets.^25–28^ These models reduce the need for system-specific quantum data and have shown strong performance across many small-molecule and materials benchmarks. However, due to their recency, their behavior for macromolecular systems remains largely untested. Moreover, even when UMLIPs provide a reliable baseline, fine-tuning or system-specific adaptation is often needed for application-specific studies. Thus, understanding which molecular environments must be represented is still quintessential, regardless of whether one uses a bespoke MLIP or a UMLIP.

In this work, we provide a control study of this question. Using linear alkanes with *n* = 1–8 as a model system, we examine when MLIPs trained exclusively on small molecules can accurately extrapolate to longer chains and more complex architectures. By combining MLIP performance analysis with environment-level descriptors and classification tools, we identify the aspects of the molecular environment distributions that govern extrapolation accuracy and show how neighbor-list design influences the learnability of inter- versus intramolecular interactions. These results demonstrate the principles behind MLIP extrapolability and constitute a proof of concept for building transferable MLIPs for macromolecular systems in a way that complements, and in some cases extends, the capabilities of traditional force-field approaches.

## Methods

### Data Generation

#### Training Sets

Each MLIP was trained exclusively on a single *n* = 1–8 alkane data set. To limit confounding factors in our analyses, data sets were constructed at similar phase, pressure, and temperature conditions. Each data set consisted of *N* = 64 molecules, simulated at *T* = 300 K with the volume corresponding to NIST fluid properties’ density for each alkane at 5 MPa and 300 K.^29^ At these temperature and pressure conditions, each alkane is in a liquid state, aside from methane, which is a supercritical liquid. Initial configurations of *n* = 3–8 alkanes were obtained from the Cambridge Structural Database.^30^ For methane and ethane, initial configurations were generated using RDKit^31^ on a cubic lattice with the appropriate volume. Each initial configuration was relaxed to an unordered, liquid state using isobaric–isothermal (NPT) molecular dynamics simulations at low pressure (*P* = 0.5 MPa, *T* = 300 K). These NPT simulations were performed with a Berendsen barostat and thermostat, a time step of 0.2 fs as well as periodic boundary conditions, and were run until the reference experimental density from NIST was obtained and energies had converged. These convergence thresholds were set to ensure that the underlying energetics would reasonably reflect the experimentally relevant systems, thereby avoiding under- or overestimating intermolecular contributions based on the chosen energetic model (density functional tight binding (DFTB)).^32,33^

For *n* = 7, 8 data sets, further relaxation at large box volume (with *P* = 0.01 MPa, *T* = 300 K) and geometry optimization were necessary in order to remove initialization artifacts. Initial configurations had metastable ordering due to finite size effects. After these systems were randomized, we repressurized these two data sets to 300 K and 5 MPa with the OPLS-AA force field until they reached the appropriate volume^34^ using periodic boundary conditions, a time step of 1 fs, and a Nosé–Hoover thermostat and barostat. Initial velocities were generated using Maxwell–Boltzmann sampling at 300 K without fixing a random seed. With *n* = 8, some initialization artifacts still remained, so we computed a structure relaxation in ASE using a Broyden–Fletcher–Goldfarb–Shanno (BFGS) algorithm and *f*_max_ = 0.05 with a DFTB+ calculator using a conjugated gradient driver to anneal the system.

After the appropriate box volumes were reached, NVT simulations were then conducted at 300 K. These production simulations were conducted using DFTB+, initially using a grid of (1, 1, 1) k-points with a Berendsen thermostat, an initial stochastic Maxwell–Boltzmann velocity distribution, a time step of 0.2 fs, as well as periodic boundary conditions, and were run until potential energies had converged. For our purposes, an equilibrium cutoff was defined as where the running average of the total energy did not fluctuate by more than 0.1 eV for 2,000 consecutive frames in the NVT simulation. After this period, we ran systems for at least an additional 100,000 timesteps. An example DFTB+ script is provided in the Appendix. We then selected 1000 frames from these “production runs” at random to form testing sets and used farthest-point sampling^35–37^ on smooth overlap of atomic positions (SOAP^6^) vectors to select a mutually exclusive 10,000 frames to constitute our training sets. After sampling, we recalculated the potential energies and forces for these frames using a grid of (3, 3, 3) k-points. This choice of energetic model is motivated by the chemical simplicity of alkanes, as DFTB has generally been shown to provide a reasonable description of both intra- and intermolecular interactions in systems with small dipoles and saturated sp^3^ bonding.^32,33^ Nonetheless, we acknowledge that intermolecular energetics can vary across levels of electronic structure theory, and we therefore cannot rule out some quantitative changes under higher-level references.

#### Additional Testing Sets

Decane, dodecane, cyclohexane, 4-propylheptane, and 3,3-diethylpentane configurations were sampled using the OPLS all-atom (AA) force field.^34^ Decane and dodecane were simulated with 48 molecules, and the others with 64 molecules, in order to keep computational overhead consistent. The molecules were initialized on a three-dimensional lattice with 10 Å spacing between molecules and with each dimension of each lattice position randomly perturbed by −5 to 5 Å sampled from a uniform distribution. We equilibrated the systems in an NPT ensemble with respect to the average radius of gyration, which quantifies the average size of the alkane conformations. The radius of gyration of an alkane, *R*_g_, is given by $R_{g}=\sqrt{\frac{\sum_{i}m_{i}r_{i}^{2}}{\sum_{i}m_{i}}}$ , where the sums are over atoms *i* in the alkane, *m*_*i*_ is the atomic mass, and *r*_*i*_ is the displacement relative to the alkane center of mass. We used pyMBAR^38^ to calculate the correlation time of the radius of gyration time series. We equilibrated each system for 100 correlation times. Figure S1 demonstrates the equilibrium radius of gyration of the alkanes. Once equilibrated, the systems were run for 1 ns. All classical simulations were run in LAMMPS^39^ using force field parameters generated by ligpargen^40–42^ in an NPT ensemble at 300 K and 5 MPa using a Nosé–Hoover thermostat and barostat with loose couplings of 1 ps^–1^ each and a time step of 1 fs. We then selected 100 decorrelated frames from each trajectory and recomputed energies and forces using DFTB+.

#### Energy and Force Evaluations

For all frames in the training and testing data, we recomputed the energies and forces with DFTB+ using self-consistent charges and the mio Slater–Koster files^43^ using a Monkhurst–Pack grid-point interpolation (3, 3, 3). We utilized a high-throughput workflow with signac^44^ and signac-flow.^45^

To construct values for inter- and intramolecular energies and forces, we isolated the individual molecules into separate ASE atoms objects and recomputed energies and forces without periodic boundary conditions to prevent single molecules from interacting with their image. This yielded

$$E_{\mathrm{intermolecular,}\mathrm{bulk}}=E_{\mathrm{total,}\mathrm{bulk}}-E_{\mathrm{intramolecular}}$$ 1

where *E*_intramolecular_ ≡ ∑_*i*=1_^64^ *E*_*i*_, with *E*_*i*_ the total energy of the *i* ^th^ single molecule.

### Machine-Learned Potentials and Analyses

#### MACE MLIPs

For every MLIP, we solely used the aforementioned training sets during construction and trained each model as one normally would for a nonextrapolative task. When training solely on intramolecular quantities (Figure S4), we down-selected from the 640,000 single-molecule frames to 10,000, choosing one random molecule from each training frame. Testing sets were kept consistent, with 64,000 single molecule frames in total.

The MACE^7^ model (higher-order equivariant message passing neural network) combines equivariant graph neural network design with explicit many-body message passing. Unlike most standard MPNNs that rely on only two-body (pairwise) interactions and require many message-passing layers to build complexity, MACE constructs higher-body (up to four-body) equivariant messages in a hierarchical fashion. This allows the network to achieve high representational power with just two message-passing iterations, substantially reducing depth, improving parallelism, and maintaining scalability while reaching or surpassing state-of-the-art accuracy on benchmarks such as rMD17, 3BPA, and AcAc.^7^ By building hierarchical tensor products of lower-order features and symmetrizing via generalized Clebsch–Gordan coupling, MACE efficiently implements these many-body messages without exponential costs. This induces equivariant feature tensors, which are linearly combined (with weights dependent on element type and irreducible representation indices) to produce message updates. For our MACE-based potentials, we use the software v.0.3.13 and a standard set of model hyperparameters, as shown in the example script in the Appendix.

#### SOAP Analysis

Smooth Overlap of Atomic Positions (SOAP) vectors were computed using the featomic^46^ and librascal^47^ packages with an interaction cutoff of 10 Å (for Figures 2 and S5) and 7 Å (for Figures 3 and S6). In all cases, we used the GTO basis set with 8 radial components and 4 angular components. Principal Covariates Classification (PCovC,^48^) was computed using v0.3.2 of scikit-matter,^35^ using as *X* the aforementioned SOAP vectors of the training set, a mixing parameter of 0.5, a logistic regression classifier, and oligomer length *n* as our class value. Each PCovC model was computed solely for like environments, such as hydrogen, CH_2_, or tertiary carbon environments (Figures 2 and S5). Prior to fitting and analysis, feature vectors and energies were appropriately scaled and centered using scikit-matter^35^ StandardFlexibleScaler. For regression (Figure 3), we used scikit-learn^49^ RidgeCV with cv = 5 and a negative mean squared error scorer. Twenty regularization constants (α) were considered for RidgeCV using a logarithmic grid.

## Results

### Extrapolating Energies and Forces

Figure 1 shows the results of using trained MLIPs to predict the energies and forces on different oligomeric chain lengths. On first glance, the energy-fitting results of Figure 1a tells a discouraging story—each training set is unable to predict the energies of the other alkanes, with a MAE well above chemical accuracy (which we define as 1 meV/atom^50^). However, the force results (Figure 1b) imply something much different, in line with our chemical intuition—at larger chain lengths, we can reasonably extrapolate the energetics of one system to the next (and even those below).

**Figure 1:**
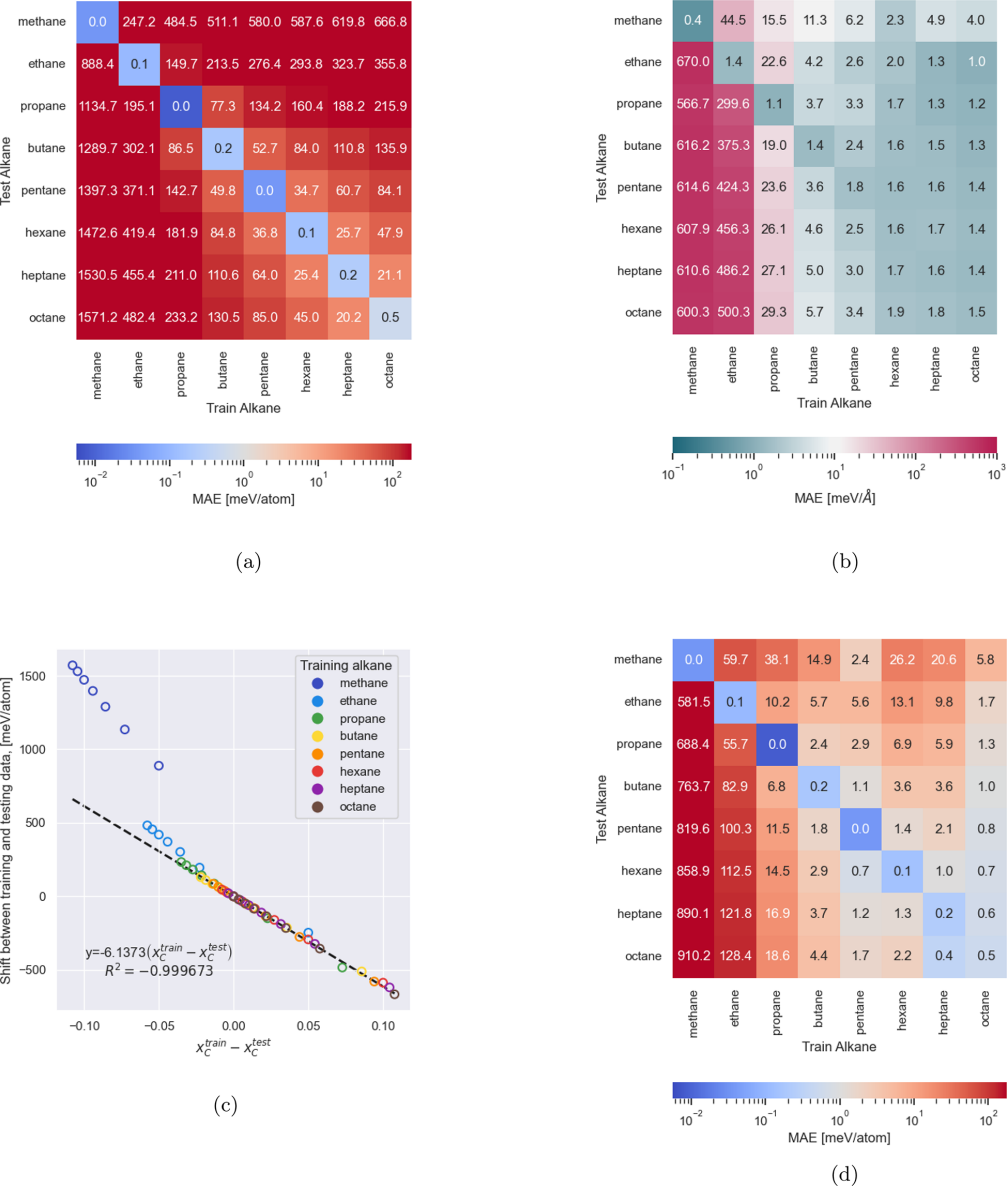
Results of MACE potential building on the total potential energy on *n* = 1–8 alkanes. (a) Results of predicting per-configuration energies (normalized by the number of atoms) using MACE MLIPs for different train (*x*-axis) and test (*y*-axis) pairings, respectively. Color indicates the mean absolute error (MAE) in units of meV/atom, respectively. (b) Results of predicting atomic force vectors using the MACE MLIPs for different train (*x*-axis) and test (*y*-axis) pairings, respectively. Errors correspond to the average magnitude of vectorial error across the testing set. Color indicates the mean-absolute-error (MAE) in units of meV/Å, respectively. (c) The relationship between the shift in composition (*x*_C_^train^ – *x*_C_^test^) and the shift in energies for extrapolated MLIPs. The models trained on the methane, ethane, and propane data sets show a divergence from this proportionality, as they are not necessarily considered “well-conditioned.” (d) Shifted energy errors for MACE energies, accounting for the learnable shift in (c).

So, why do these two results diverge? A first intuition would be data centering—machine-learning potentials are typically trained to predict the *fluctuations* in molecular energetics, not their absolute values. In other words, the majority of MLIP methods and software first subtracts the mean of the training targets before determining weights and biases. This means that the predicted energy *E*_pred_ is ostensibly

$$E_{\mathrm{pred}}=E_{\mathrm{MLIP}}+b$$ 2

where *b* is a “baselining constant” or the energetic values shared by all training points. This is verified by the strong colinearity between our predicted and true values, as shown in Figure S3. A simple explanation of this “mean-shift” would be the shift in atomic energies

$$E_{\mathrm{atom}}=n_{C}E_{C}+n_{H}E_{H}=N(x_{C}E_{C}+x_{H}E_{H})$$ 3

which, when learning per-atom energies *E*/*N*, would yield

$$b≡(x_{C}^{\mathrm{train}}-x_{C}^{\mathrm{test}})(E_{C}-E_{H})$$ 4

However, this is not the case here, where a shift due solely to atomic contributions would lead to a much larger value than the observed shift. This presents an issue for extrapolating energetics from one system to another, where the only information known a priori may be the difference in underlying chemistry and where “transfer learning” may be infeasible due to the lack of underlying training data. Thus, we first determine if *b* is a linear function of our composition (*x*_C_, *x*_H_), as this would still allow for the extrapolation of one system to another without prior computation of energetics or configurations.

For models trained on *n* = 4–8 alkanes, there exists a strong linear proportionality (Pearson correlation coefficient = 0.9997, Figure 1c) between the compositional shift and the mean-shift of the data sets. This suggests that these mean-shifts are learnable parameters, even if not obviously grounded in physical values such as *E*_atom_. To test this, we determined a slope to the line in Figure 1c via nonregularized linear regression, omitting from training the values obtained from models built on methane (blue), ethane (light blue), and propane (green), which are considered unsuccessful models. Accounting for the large colinearity of the mean-shift and compositional change, many of the error predictions are improved, yet there exist some residual errors (Figure 1d). This suggests that the change in conformational distribution contributes non-negligibly to the mismatch in energies.

However, the results of Figure 1b still demonstrate that there exist two critical lengths of alkanes for extrapolation: (1) butane, where we see a general reduction in force error magnitude from 20 to 30 meV/Å to 3–6 meV/Å (omitting methane) and (2) hexane, after which we see little improvement in force error with additional molecular units. The change in errors from propane to butane follows our chemical intuition—prior to this chain length, there is no sampling of the dihedral rotation possible in these molecules, and thus any system (*n* = 4–8) that contains these interactions would be ill-represented by such models.

But what makes hexane special? To understand this, we can look at the higher-dimensional representation of the interaction environments shared and distinct within the training sets. While we could arguably use the learned features of the MACE MLIPs, we instead employ a preceding, related technology, the SOAP,^6^ which is deterministic in its description of atomic environments without the implications of training on one data set versus another. Both MACE and SOAP vectors compute the many-body expansion of atomic interactions, and thus we can infer some behaviors of MACE based on a SOAP representation. We construct our SOAP descriptors based on the distance of the MACE perceptive field (10.0 Å).

After we compute the SOAP vectors for each training set, a standard analytical task is to look at the principal components analysis (PCA) of all vectors, which would demonstrate the diversity of environments. However, here we are particularly interested in how the environments within these data sets differ, which is not the underlying mathematical motivation of PCA, and this aspect can be largely lost in PCA (and other unsupervised) maps. So, we instead employ hybrid supervised-unsupervised analyses called PCovC,^35,48,51,52^ which computes the two-dimensional projection that both retains PCA-like variance and best separates the data based on assigned labels (here the number of carbons in the data set backbone). Here, we focus on the three-body interactions of the CH_2_ terms, which demonstrate the greatest statistical difference from data set to data set, with PCA and PCovC maps of remaining environments in Figure S5.

From this analysis, shown in Figure 2, we see that the convergence in MLIP error corresponds with the sampling of the CH_2_ environments three carbons from the end (first present in hexane), and the addition of further internal environments within heptane and octane leads to diminishing returns in terms of force approximation. This demonstrates that determining the minimal chain length required for MLIP construction (or molecular fragmentation^22–24,53^ ) amounts to determining the convergence of the majority of molecular environments, a task that can be accomplished via classification models or similar unsupervised tasks on single-molecule data.

**Figure 2:**
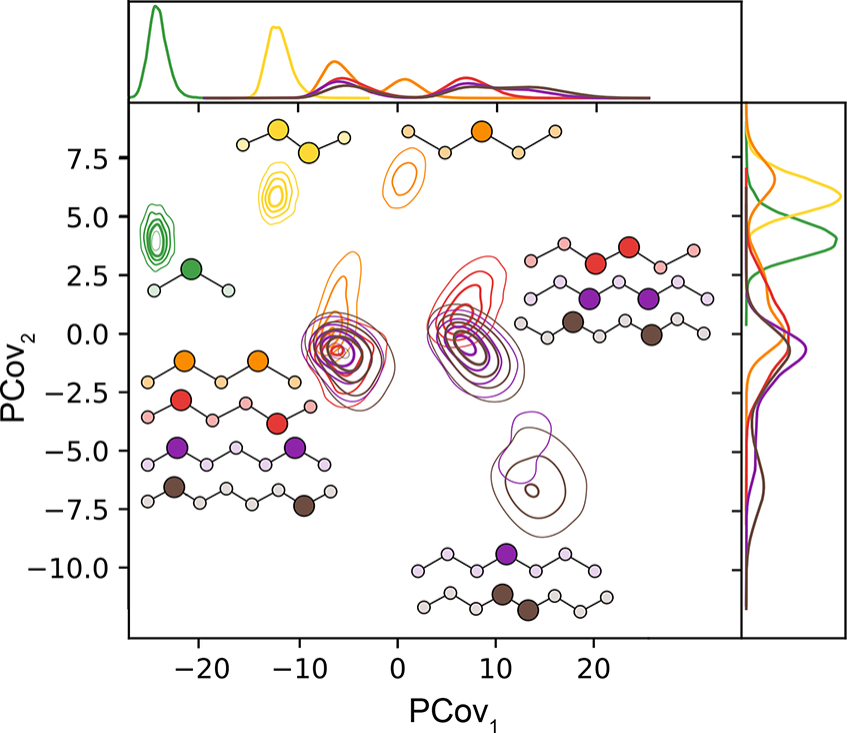
Distribution of CH_2_ environments for the different training sets, mapped via PCovC.^48^ The axes are directions in latent space that demonstrate the highest degree of diversity in the CH_2_ environments and separability between the data sets. Points that are overlapping are considered “indistinguishable,” in that an ML model could not tell whether the environment sits in one alkane length or another. The map demonstrates the convergence of environments with increasing *n*. Different alkanes are denoted in different colors (green–propane, yellow–butane, orange–pentane, red–hexane, purple–heptane, and brown–octane), with the corresponding CH_2_ environment enlarged for each cluster in the map. The plots on the side opposite the axes represent the marginal distributions of each training set along the opposite axis.

While previous work has emphasized the ability of diverse, mixed chemical training data sets to increase the accuracy of MLIPs for hydrocarbon systems and to enable UMLIP development,^25–28,54^ our analysis of MLIPs trained exclusively on individual alkane data sets allows us to directly relate the convergence in force and energy errors with the convergence in the underlying chemical environments in training data sets. As a result, we can clearly see how the emergence of internal CH_2_ environments in hexane forms a minimal spanning basis governing extrapolation behavior among *n* = 1–8 alkanes. When shorter chains are used as training data, extrapolation becomes less feasible, both in terms of the linear shift in energies as a function of composition (Figure 1c) and the lack of overlap in configurational distributions (Figure 2). We will further examine the performance of these MLIPs on more structurally complex hydrocarbon systems to better understand extrapolative behavior in a later section.

We note that the intuitive notion that similar chemical environments should have similar parametrization is a key underlying principle of classical force fields.^3^ These methods use different sets of parameters for the same atoms depending on their local environment, as demonstrated in topological methods such as TAFFI^55^ or string-based representations in SMIRNOFF.^56^ Here, although we specify only the element of each atom, the MLIP is able to naturally cluster the relevant local environments without human input. This is possible for MLIPs because the representation learned during training naturally distinguishes between different local environments for the same chemistry. This presents a key advantage over classical approaches in terms of their accuracy and ease of use.

### Separating the Different Contributions to Total Energies

However, predicting the total energies and forces may not be sufficient for a usable potential model, as the hierarchical nature of interactions within these systems decides much of their fundamental behavior.^57^ Conceptually, and in classical force fields, we consider potential energies emerging from three distinct classes of interactions:

$$E=E_{\mathrm{atom}}+E_{\mathrm{intramolecular}}+E_{\mathrm{intermolecular}}$$ 5

as these terms have significantly different magnitudes, difficulties in calculating, and difficulties in terms of “learnability.” *E*_atom_ is the largest component but arguably trivial to compute. We can group *E*_atom_ with intramolecular interactions to obtain an *Ẽ*_intramolecular_ = *E*_atom_ + *E*_intramolecular_ by sampling single-molecule configurations; this is, likewise, a large component of *E*. As shown in Figure S4, extrapolating *Ẽ*_intramolecular_ follows the same lessons learned in predicting *E*—both in terms of the necessary molecule lengths for extrapolation and the ML baselining leading to divergent narratives for energy- and force-fitting.

The element of this energetic expansion that requires the greatest nuance is the *intermolecular* interactions *E*_intermolecular_, which are comparatively weak, longer-range, and computationally expensive to calculate using methods from quantum chemistry.^58^ When training on total energies, the resultant errors often overshadow these small contributions,^57^ losing interactions that are decisive in many critical scientific problems.

MACE itself (v.0.3.13) is not natively suited for directly learning these interactions, nor are atom-centered expansions that implicitly or explicitly focus on the nearby atoms in constructing their MLIP.^59^ To demonstrate this, we again turn to the SOAP^6^ framework and show how careful attention to weighting interactions can yield better fidelity for intermolecular models. Following the same procedures in terms of training/testing splits, we construct cross-validated ridge regressions on SOAP representations for the same *n* = 1–8 alkane data sets used for MACE MLIP construction. SOAP vectors were calculated with a cutoff of 7 Å for atomic environments and averaged for each frame such that for the 10,000 training frames and 1,000 testing frames, we have 10,000 and 1,000 corresponding SOAP vectors; we refer to these initial SOAP calculations as the “total” SOAP vectors or *X* ^total^.

Similar to the initial results from MACE in Figure 1a, the results of using a *E*_intermolecular_ ≈ *X* ^total^ *w*, shown in Figure 3a, are discouraging. Extrapolation, with a field-standard threshold MAE of 1 meV/atom,^50^ is only achieved three times (by hexane on heptane and heptane on hexane/octane), despite the considerably smaller magnitude of the intermolecular energetics, as compared to the total energies regressed earlier. However, we can adopt lessons from learning the lattice energy of molecular crystals^59^—SOAP representations, and representations that are implicitly or explicitly “near-sighted” ^7,60,61^ will, by design, consider the contributions of nearby atoms more strongly than those further away. This will overshadow the intermolecular environments which contribute to *E*_intermolecular_, limiting our accuracy in extrapolation.

**Figure 3:**
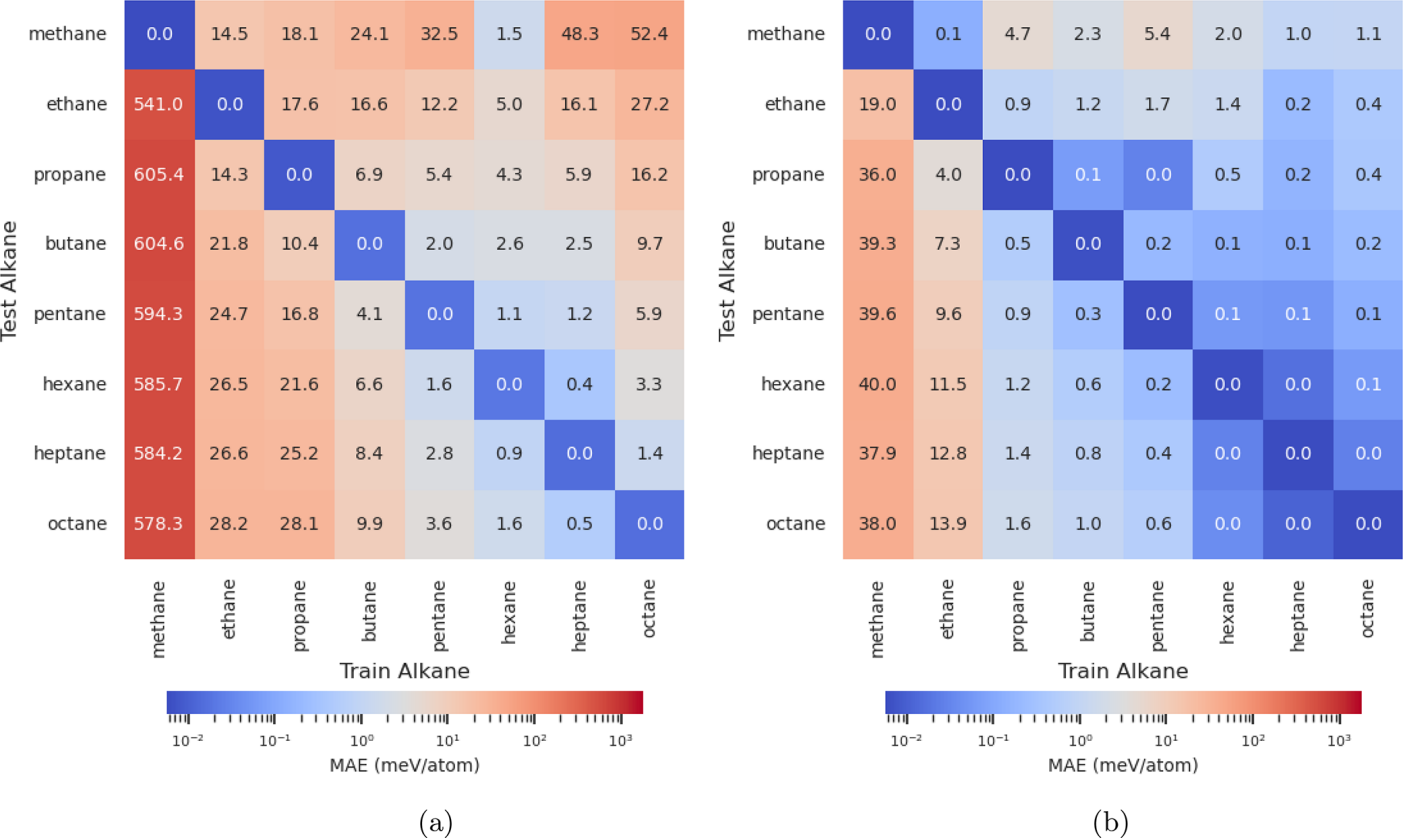
Results of SOAP-Ridge MLIPs trained on intermolecular potential energy using different SOAP representations. (a) Regressions using total SOAP vectors *X* ^total^. (b) Regressions using the far-sighted SOAP vectors *X* ^fs^.

We can thus construct a “far-sighted” SOAP vector

$$X^{\mathrm{fs}}=X^{\mathrm{total}}-\frac{1}{64}\overset{64}{\underset{i=1}{\sum }}X_{i}$$ 6

where *X*_*i*_ is the averaged SOAP vector of the atomic environments in the *i* ^th^ molecule within the bulk frame. Effectively, this representation reweights the feature space by reducing (or eliminating) the influence of contributions from intramolecular environments on the SOAP vector. As shown in Figure 3b, using this far-sighted representation results in a significant improvement in the number of extrapolative regimes achieved by SOAP-Ridge MLIPs in comparison to the results in Figure 3a. Similar trends are observed as in Figure 1b: steep improvements in MAEs are observed between ethane and propane/butane, and once again from pentane to hexane. This follows the convergence in chemical environments (Figures 2 and S5). Furthermore, these results demonstrate that careful consideration of MLIP features can improve performance for targets other than the total potential energy of a system.

### Beyond *n* = 1–8 Linear Alkanes

It is worth inspecting the limitations of these models when considering longer molecules or more complex hydrocarbon architectures. As shown in Figure 4a, decane (*n* = 10) and dodecane (*n* = 12), the trends follow those similar to the shorter oligomers within the series: we see a reduction in error when moving from propane to butane (30 meV/Å to 5–6 meV/Å), and a saturation near 1.5–2 meV/Å once training on hexane through octane. This supports our earlier observations considering the saturation of interaction environments and suggests that these trends should continue to longer linear alkanes. Returning to the SOAP-Ridge MLIPs constructed using total SOAP vectors (*X* ^total^) and far-sighted SOAP vectors (*X* ^fs^) in Figure 4c, we also maintain previous trends: intermolecular energy MAEs decrease consistently as the training alkane chain length increases, and MLIPs constructed using *X* ^fs^ consistently outperform MLIPs constructed with *X* ^total^. This reconfirms our earlier claim that reweighting the feature space to exclude intramolecular contributions enables higher accuracy when targeting intermolecular energy.

**Figure 4:**
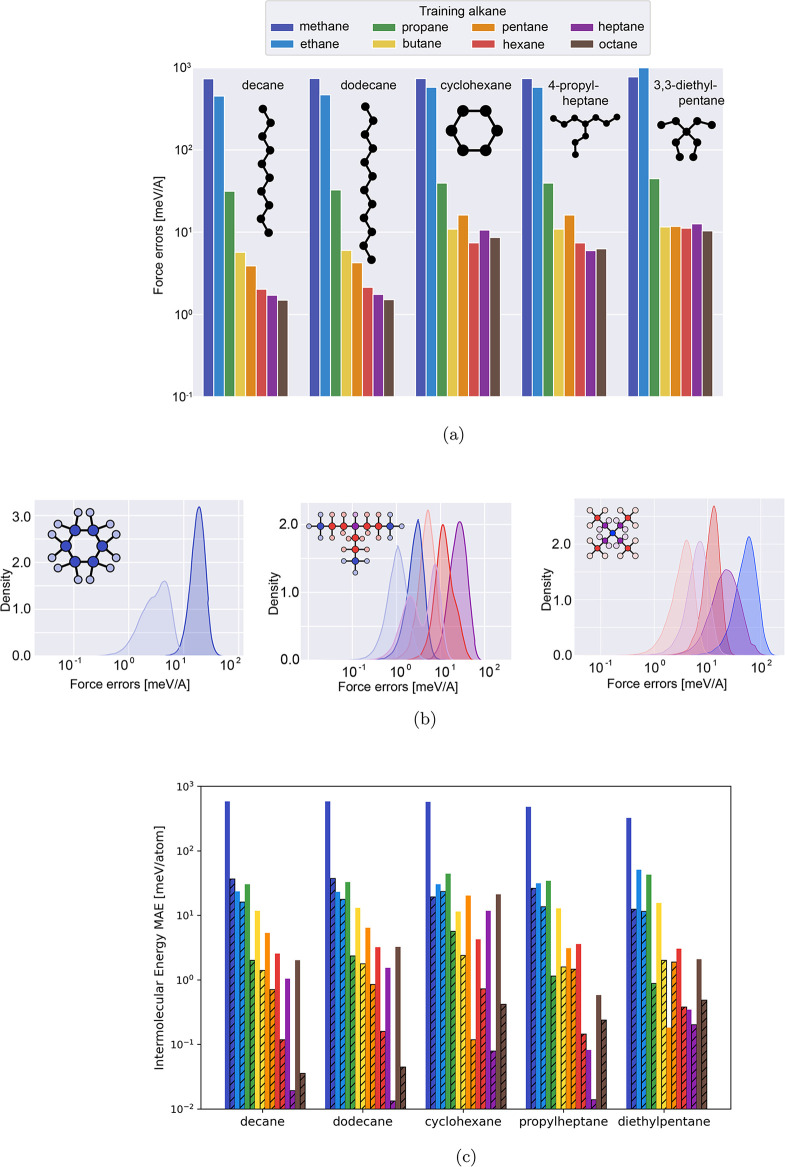
Force and energy errors for decane, dodecane, and the nonlinear alkanes. (a) Force errors resulting from testing *n* = 1–8 trained MLIPs on longer chain lengths and architectures. Color of the bar plot denotes the training set used, and each set of bars refers to a different test molecule. (b) Distribution of force errors for cyclohexane (left), 4-propylheptane­(center), and 3,3-diethylpentane (right), as predicted by a model trained on octane. Colors indicate the atoms within the molecule. For every carbon, the corresponding hydrogens are in the same hue, with a lighter tint. (c) Intermolecular energy errors resulting from testing *n* = 1–8 trained SOAP-Ridge MLIPs on longer chain lengths and architectures. Color of the bar plot denotes the training set used, and each set of bars refers to a different test molecule. Hatched bars indicate MLIPs constructed using far-sighted SOAP vectors *X* ^fs^; solid bars indicate MLIPs using total SOAP vectors.

Molecules of different architectures fare less well in extrapolation. For cyclohexane, 4-propylheptane, and 3,3-diethylpentane, we typically converge to higher values (6–10 meV/Å) than within the linear series (1–2 meV/Å). On one hand, this is due to the lack of longer alkyl chains within cyclohexane, 4-propylheptane, and 3,3-diethylpentane—moving to hexane, heptane, and octane training data is not nearly as beneficial when the longest uninterrupted alkyl chain is three carbons long. For 4-propylheptane and 3,3-diethylpentane, the larger errors are largely attributed to the tertiary and quaternary carbons (Figure 4b), neither of which exists in a linear alkane. Though we see higher errors from the MACE-predicted forces for these molecules, we maintain consistent trends in intermolecular energies as predicted by SOAP-Ridge MLIPs with decreasing errors as training alkane chain length increases, and lower errors for all but one far-sighted MLIPs.

Cyclohexane presents a more interesting case. The carbons in cyclohexane have long received different parametrization than those in hexane via popular force fields such as OPLS-AA.^34,62,63^ This has largely been attributed to the constrained torsion angles in cyclohexane, which remove the freely rotating C–C dihedrals that characterize linear alkanes.^62^ However, here, the key difference is the local environment distribution: linear alkanes provide very few CH_2_ environments with neighboring CH_2_ at the 2–3 Å range, whereas cyclohexane contains several such neighbors because of its compact ring geometry. As a result, when the model trained only on octane is evaluated on cyclohexane, it encounters CH_2_–CH_2_ configurations that were poorly sampled in the training set, producing the shift observed in Figure 4b.

## Conclusions

In this study, we have demonstrated key behaviors of MLIPs that can be applied to build predictive models for oligomeric systems, with implications for MLIP extrapolation for long-chain and polymeric materials. First, we demonstrate and visualize that, in systems with similar chemistries, extrapolation of forces and energies *is* possible, provided that those interactions present in the target molecule are properly sampled within the smaller analogues. Specifically for alkanes, we see a strong reduction in error with butane and we see the force errors converging once we reach hexane. These results were also demonstrated for longer linear alkanes and different architectures. The latter proved to be more nuanced and complicated as an extrapolation task; while nonlinear alkanes contain the same local bonding, MLIPs do not consider these aspects explicitly but rather as emerging from data distributions. Because the environmental distributions of branched or cyclical alkanes are fundamentally different from linear alkanes, the MLIPs fare worse in extrapolating to these systems.

Second, we showed that extrapolation of energies results in a learnable shift between different molecules; this can be largely ameliorated by predicting the mean shift in probability distributions as a function of the composition. As forces are not affected by constant shifts in energy, the extrapolation of forces between different molecules is straightforward and does not require the accounting for this effect.

Finally, we demonstrated a key finding about the hierarchy of energies. Intermolecular contributions are important in determining thermodynamic properties but have historically been challenging to predict due to their small magnitude relative to the intramolecular component. We have shown that careful partitioning of molecular descriptors can yield MLIPs capable of predicting and more reliably extrapolating intermolecular energetics. This suggests that some of the most costly calculations, those relating the interactions of one molecule to another, can be translated from one system to another of similar chemistry after performance has converged in terms of the chain length.

The conclusions of this study also inform the larger context of MLIP development, particularly the introduction of universal or ”foundational” machine-learned interatomic potentials (UMLIPs) that have recently emerged as a promising direction, offering broad chemical coverage from large, diverse training sets.^25–28^ These models reduce the need for system-specific quantum data and have shown strong performance across many small-molecule and materials benchmarks. However, even when UMLIPs provide a reliable baseline, fine-tuning or system-specific adaptation is often needed for application-specific studies. Thus, understanding what our “minimal data set” required is still quintessential.

## Supplementary Material



## Data Availability

The data sets generated and/or analyzed during the current study are available in the Zenodo repository at Hooven et al.^64^ [DOI: 10.5281/zenodo.17227874]. The underlying code used in this study are available via open-source software through GitHub, namely MACE (https://github.com/ACEsuit/mace^7^), scikit-matter (https://github.com/scikit-learn-contrib/scikit-matter^52^), featomic (https://github.com/metatensor/featomic^46^), and librascal (https://github.com/lab-cosmo/librascal.git^47^).

## Notes

Additional methodological details and supporting figures (PDF)
