# Supplementary material for: How Long Is Long Enough? Extrapolation of Machine-Learning Interatomic Potentials for Oligomeric and Polymeric Systems
Source: J Chem Theory Comput. 2026 Jun 19;22(13):6917–26. doi: 10.1021/acs.jctc.6c00365 (PMC13374000; doi:10.1021/acs.jctc.6c00365)
Supplement: Supplementary file 1 [file ct6c00365_si_001.pdf]

How Long is Long Enough? Extrapolation of Machine-Learning Interatomic Potentials for Oligomeric and Polymeric Systems

Natalie E. Hooven[1], Arthur Y. Lin[1], Charles H. Carroll[1], and Rose K. Cersonsky*[1,2,3]

[1]Department of Chemical and Biological Engineering, University of Wisconsin - Madison, Madison, WI, 53706, USA
[2]Department of Materials Science and Engineering, University of Wisconsin - Madison, Madison, WI, 53706, USA
[3]Data Science Institute, University of Wisconsin - Madison, Madison, WI, 53706, USA

*Email: rose.cersonsky@wisc.edu

Additional methodological details

NVT DFTB+ production simulations of $n = 1 - 8$ alkanes were performed using the following script (units in atomic units). Note that after equilibration was reached in production runs and sampling performed, frames were recalculated on a grid of (3, 3, 3) k-points.

Listing S1: Sample dftb_in.hsd file used for NVT DFTB+ production simulations.

```
Geometry = GenFormat {
    <<< "geo_end.gen"
}

Driver = VelocityVerlet{
   KeepStationary = Yes
   MDRestartFrequency = 1
   Periodic = Yes
   Steps = 100000
   Thermostat = Berendsen{
      CouplingStrength = 0.01
      Temperature = 0.000950044603573917
   }
   TimeStep = 8.26
   }
Hamiltonian = DFTB{
   KPointsAndWeights = SupercellFolding {
      1 0 0
      0 1 0
      0 0 1
      0.0 0.0 0.0
   }
   MaxAngularMomentum = {
      C = p
      H = s
   }
   SlaterKosterFiles = Type2FileNames{
      Prefix = ./mio-1-1/
      Separator = "-"
      Suffix = ".skf"
      }
   }
Options {
   WriteResultsTag = Yes
   }
ParserOptions {
   IgnoreUnprocessedNodes = Yes
   ParserVersion = 1
}
```

MACE MLIPs were constructed using the following command and MACE (v.0.3.13):

Listing S2: Training script for MACE MLIPs

```
raw_dir=$(python -c "import mace; print(mace.__file__)"); dir=${raw_dir:0:-12}
python $dir/cli/run_train.py \
    --name="MACE_model" \
```

```
--train_file="$1" \
--valid_fraction=0.05 \
--config_type_weights='{"Default":1.0}' \
--E0s='{1:-6.492647589968434, 6:-38.054950840332474}' \
--model="MACE" \
--hidden_irreps='64x0e + 64x1o' \
--r_max=5.0 \
--batch_size=1 \
--max_num_epochs=1500 \
--energy_key="energy_eV" \
--forces_key="forces" \
--charges_key="charges" \
--amsgrad \
--restart_latest \
--device=cuda \
--patience=20 \
--default_dtype="float32" \
```

SOAP vectors for SOAP-Ridge MLIPs were computed using the following hyperparameters with librascal (v.0.0.1):

Listing S3: Hyperparameters of SOAP vectors for SOAP-Ridge MLIPs

```
{
    "interaction_cutoff": 7,
    "max_radial": 8,
    "max_angular": 4,
    "gaussian_sigma_constant": 0.3,
    "gaussian_sigma_type": "Constant",
    "cutoff_smooth_width": 0.5,
    "radial_basis": "GTO",
    "cutoff_function_type": "RadialScaling",
    "cutoff_function_parameters": {
        "rate": 1.5,
        "exponent": 3.0,
        "scale": 2.0
    }
}
```

Supporting figures to the text

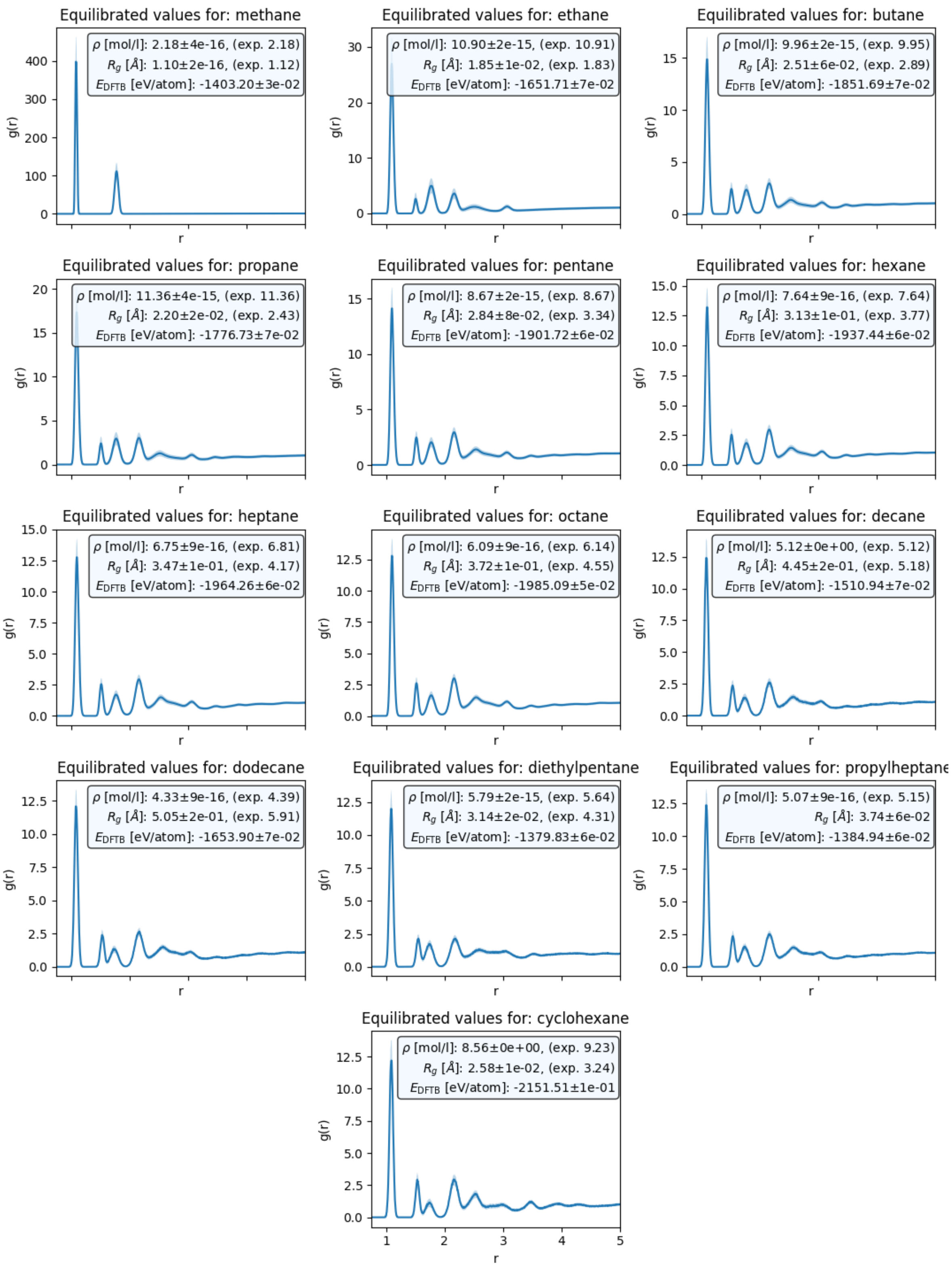

Figure S1: The converged RDFs, densities, radius of gyration, and energies for all training data and additional test sets. Relevant experimental values were obtained from the NIST thermophysical database[29] and from Yaw's Handbook of Thermodynamic Data[65]. Experimental radii of gyration were available for 1atm, which led to considerably higher values than those observed at 5MPa (this study).

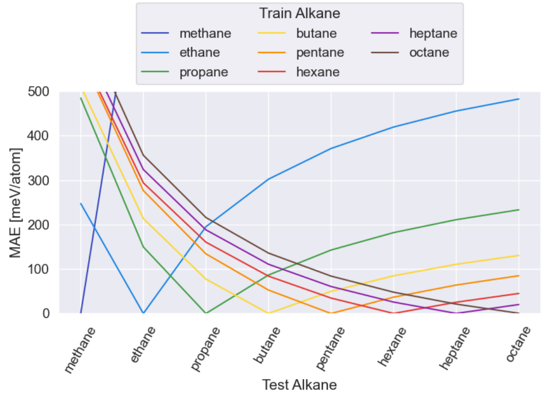

(a) Alternative representation of Fig. 1a.

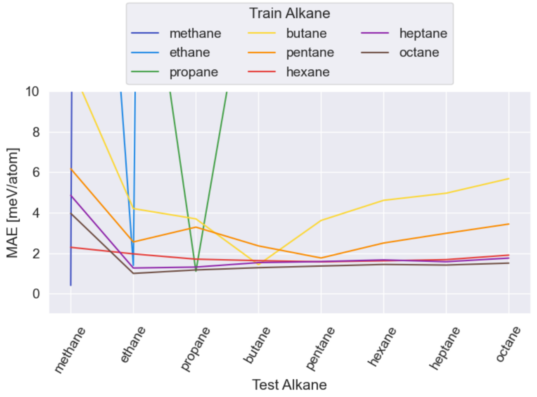

(b) Alternative representation of Fig. 1b.

Figure S2: Additional visualizations of MACE models to predict total energy and forces.

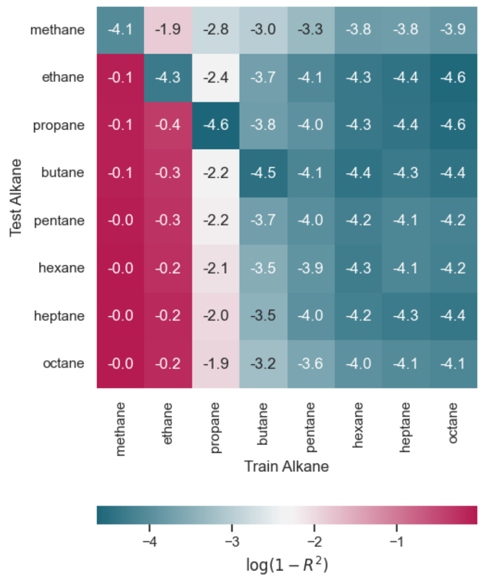

Figure S3: Map of the coefficient of determination between true and predicted values (represented as the logarithm of $1 - R^2$)

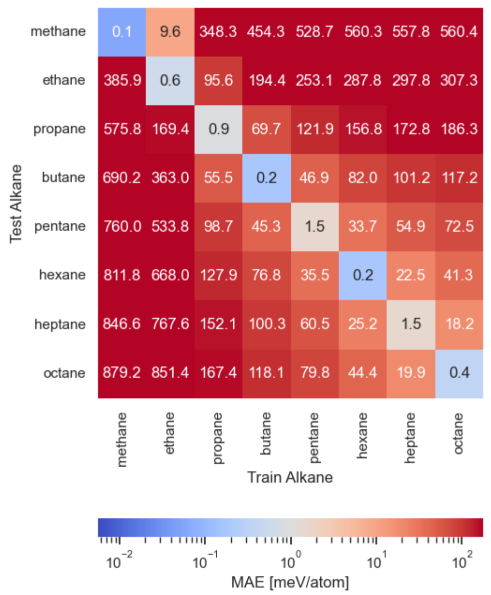

(a) Energy-fitting results of MACE MLIP for different train (x-axis) and test (y-axis) pairings, respectively. Color indicates the mean-absolute-error (MAE) in units of meV/atom, respectively.

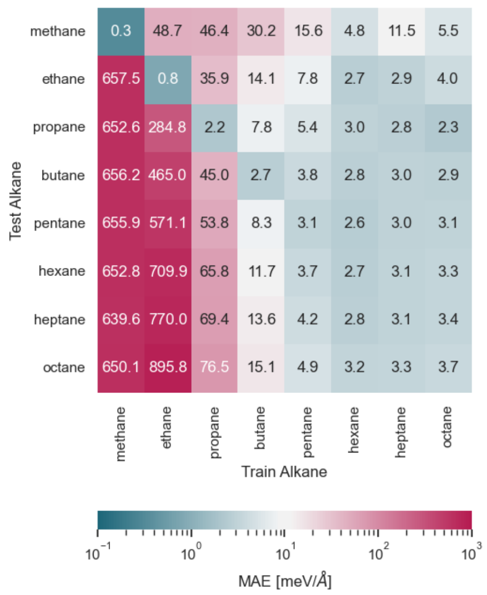

(b) Force-fitting results of MACE MLIPs for different train (x-axis) and test (y-axis) pairings, respectively. Color indicates the mean-absolute-error (MAE) in units of meV/atom, respectively.

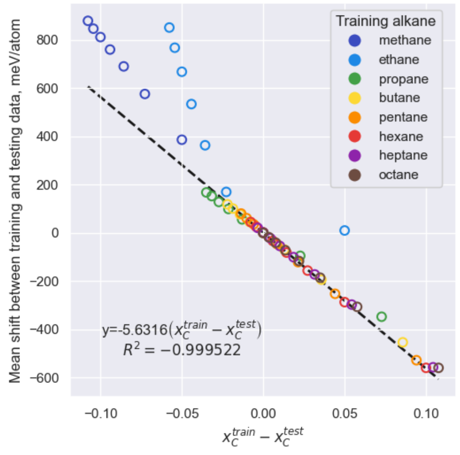

(c) The relationship between the shift in composition ($x_C^{\text{train}} - x_C^{\text{test}}$) and the shift in energies for extrapolated MLIPs. The models trained on the methane, ethane, and propane datasets show a divergence from this proportionality, as they are not necessarily considered "well-conditioned." Marker denotes the testing set, and color denotes the training set.

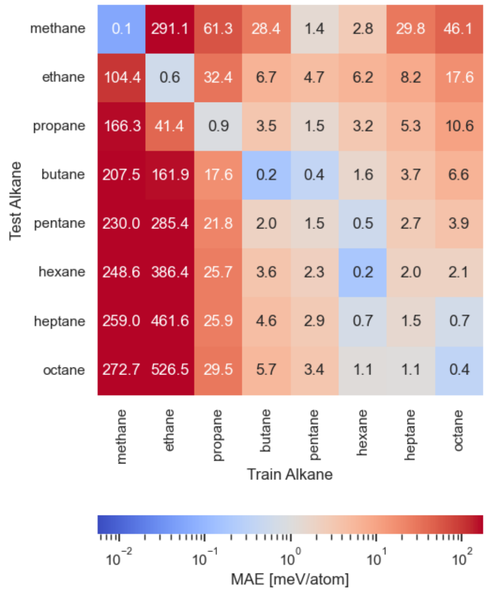

(d) Shifted energy errors for MACE energies, accounting for the learnable shift in Fig. 1c.

Figure S4: Analogous to Fig. 1, results of predicting the intramolecular potential energy ($\tilde{E}_{intramolecular} = E_{intramolecular} + E_{atom}$) on $n = 1 - 8$ alkanes.

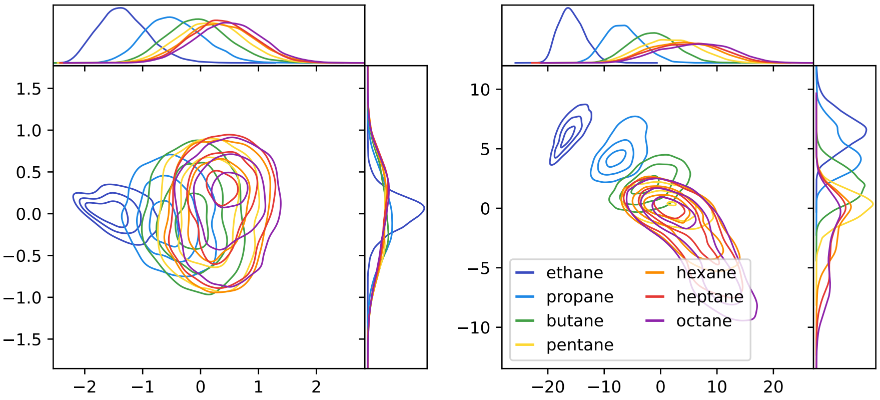

(a) Maps of hydrogen environments across first two principal components (left) or covariates (right). At a perceptive length of 10Å, we see a convergence of hydrogen environments once reaching pentane.

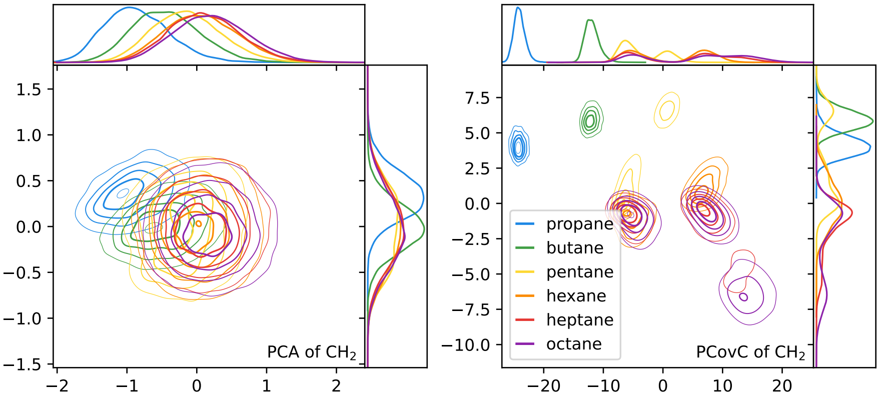

(b) Maps of CH_2 environments across first two principal components (left) or covariates (right). At a perceptive length of 10Å, we see a convergence of CH_2 environments once reaching hexane.

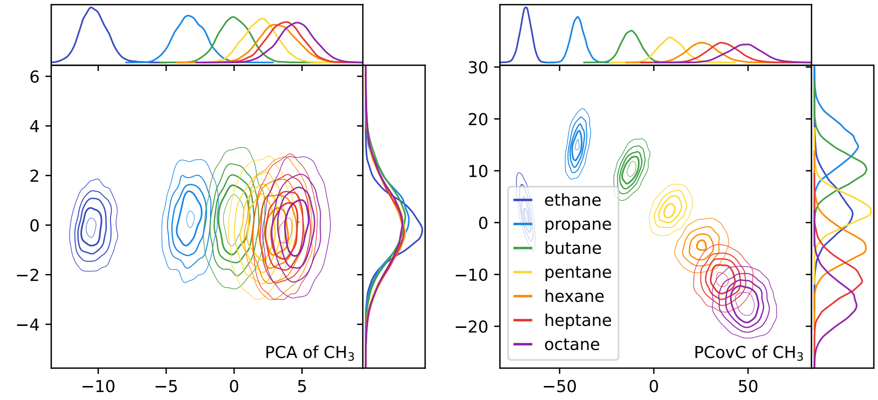

(c) Maps of CH_3 environments across first two principal components (left) or covariates (right). At a perceptive length of 10Å, we see a convergence of CH_3 environments once reaching butane.

Figure S5: Analogous to Fig. 2, maps of different environments for the different training sets, via Principal Components Analysis (left) and Principal Covariates Classification (PCovC[48], right)

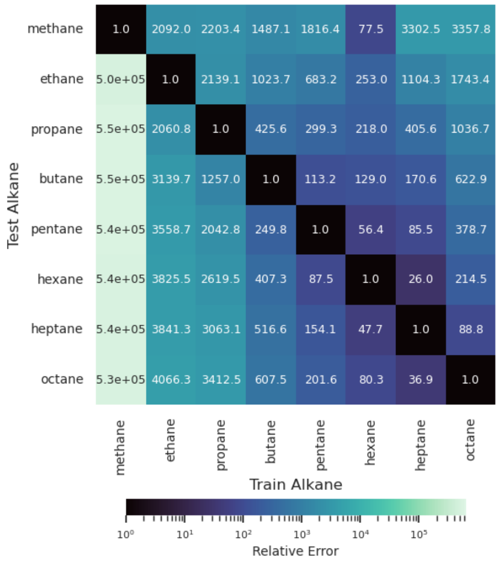

(a) Relative errors of SOAP-Ridge potentials trained on X^{total} and intermolecular energies.

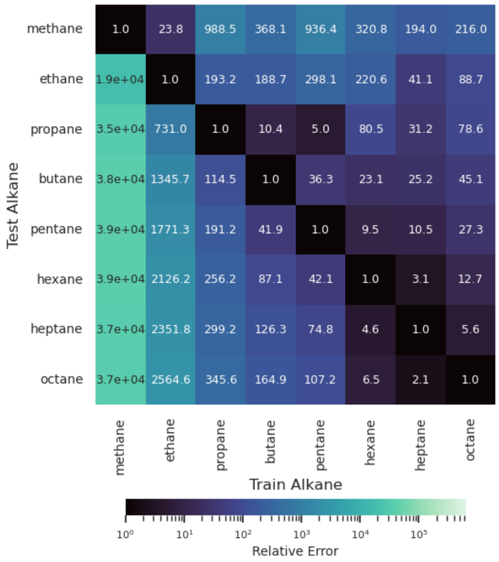

(b) Relative errors of SOAP-Ridge potentials trained on X^{fs} and intermolecular energies.

Figure S6: Analogous to Fig. 3, heatmaps of relative errors for different training and testing sets calculated using the mean-absolute-error in meV/atom for testing sets divided by the interpolative error (error of the training alkane on its testing set).
